# OSCI: standardized stem cell ontology representation and use cases for stem cell investigation

**DOI:** 10.1186/s12859-019-2723-7

**Published:** 2019-04-25

**Authors:** Yongqun He, William D. Duncan, Daniel J. Cooper, Jens Hansen, Ravi Iyengar, Edison Ong, Kendal Walker, Omar Tibi, Sam Smith, Lucas M. Serra, Jie Zheng, Sirarat Sarntivijai, Stephan Schürer, K. Sue O’Shea, Alexander D. Diehl

**Affiliations:** 1 University of Michigan Medical School, Ann Arbor, MI USA; 2 Roswell Park Comprehensive Cancer Center, Buffalo, NY USA; 3 University of Miami, Miami, FL USA; 4 Pharmacological Sciences, Icahn School of Medicine at Mount Sinai, New York, NY USA; 5 SBCNY, Icahn School of Medicine at Mount Sinai, New York, NY USA; 6 John Hopkins Unversity, Baltimore, MD USA; 7 DataSmith LLC, Detroit, MI USA; 8 Department of Biomedical Informatics, Jacobs School of Medicine and Biomedical Sciences, University at Buffalo, Buffalo, NY USA; 9 University of Pennsylvania, Philadelphia, PA USA; 10 ELIXIR, Wellcome Genome Campus, Hinxton, Cambridge UK

**Keywords:** Stem cell, Cell ontology, Cell line ontology, OSCI, iPSC, hPSC, Bipolar disorder

## Abstract

**Background:**

Stem cells and stem cell lines are widely used in biomedical research. The Cell Ontology (CL) and Cell Line Ontology (CLO) are two community-based OBO Foundry ontologies in the domains of in vivo cells and in vitro cell line cells, respectively.

**Results:**

To support standardized stem cell investigations, we have developed an Ontology for Stem Cell Investigations (OSCI). OSCI imports stem cell and cell line terms from CL and CLO, and investigation-related terms from existing ontologies. A novel focus of OSCI is its application in representing metadata types associated with various stem cell investigations. We also applied OSCI to systematically categorize experimental variables in an induced pluripotent stem cell line cell study related to bipolar disorder. In addition, we used a semi-automated literature mining approach to identify over 200 stem cell gene markers. The relations between these genes and stem cells are modeled and represented in OSCI.

**Conclusions:**

OSCI standardizes stem cells found in vivo and in vitro and in various stem cell investigation processes and entities. The presented use cases demonstrate the utility of OSCI in iPSC studies and literature mining related to bipolar disorder.

## Background

There are many resources to support stem cell research. For example, the Library of Integrated Network-based Cellular Signatures (LINCS) project [1] has a total of 38 induced pluripotent stem cell (iPSC) types, embryonic stem cell lines, and neural stem cell lines differentiated from iPSC. Approximately 230 unique batches of those cells have been used throughout LINCS assays. Initiated by the Harvard Stem Cell Institute, the Stem Cell Commons resource (http://stemcellcommons.org/) is an open source environment for sharing, processing, and analyzing stem cell data [2]. Increasingly, studies exploiting knowledge derived from stem cell data are being conducted at a large scale as exemplified in the case of the California Institute for Regenerative Medicine [3]. These examples put emphasis on the needs for robust data management for stem cell research.

Ontology plays a crucial role in data sharing, integration, and analysis by providing standardized metadata and knowledge representation. Ontology supports minimal information standards by providing formal semantics for the data elements, experimental variables, and workflow in experimental studies. Ontologies can also be used to coordinate biomedical investigations as a common terminology by providing a reference framework to foster direct comparisons of investigative findings across different experiments. The Open Biological and Biomedical Ontology Foundry (OBO Foundry) library includes over 180 ontologies that are developed under the same ontology principles and framework [4].

There are two community-based ontologies from the OBO Foundry ontology library that cover various concepts of stem cells: The Cell Ontology (CL) represents various in vivo cells, including stem cells [5]. The Cell Line Ontology (CLO) is an ontology in the domain of cell lines and the individual cell culture properties, with a focus on in vitro cell maintenance (cell line cells) [6]. Both the CL and CLO are naturally integrated as they follow the same OBO Foundry principles and framework. The representation of stem cells and stem cell lines within the CL and CLO has been developed in coordination with the needs of laboratory practice. For example, many stem cell-restricted genes have been discovered and their relationship to stem cell differentiation states needs further modeling and representation. This has given a *raison d’être* for relevant CL-CLO interactions.

The Ontology for Biomedical Investigations (OBI), co-developed by over 20 biomedical communities, covers all phases of the investigation process (e.g., planning, execution, and reporting), and the entities in these phases [7]. The OBI models general investigation variables and pipelines, which can be used and extended to represent stem cell investigation. Linking OBI to CL and CLO builds a general foundation for modeling experimental cells in the context of the work performed at bench.

The recent Workshop on Ontologies for Stem Cells and Stem Cell Line Cells (StemCellOW) (https://sites.google.com/site/stemcellow/) aimed to translate stem cell biology into an ontology framework supported by CL, CLO, and relevant OBO Foundry ontologies. This includes defining key ontology terms, ontology hierarchy design, and ontology design patterns for crucial cell processes (e.g., stem cell differentiation, replication, gene expression, and reprogramming), to support applications such as modeling of experimental use cases.

The StemCellOW workshop discussions are crystalized into two main areas of focus that will be described in this paper: (i) Development of an ontology for stem cell investigations, utilizing existing information in ontologies including CL, CLO and OBI. (ii) Applying the resulting stem cell investigation ontology concepts to a use case consisting of an iPSC study of bipolar disorder stem cell gene expression.

Bipolar disorder (BD) is a chronic neuropsychiatric condition that is characterized by unusual shifts in mood, energy, and activity levels. BD is likely to have a developmental origin as shown by altered neurodevelopmental factors in BD patient-derived neurons. The ability to reprogram adult somatic tissues into a pluripotent state now makes it possible to study the genesis of BD. Many iPSC lines from BD have been derived in several laboratories [8]. However, a better understanding of BD using iPSCs still requires careful investigation and analyses.

Deriving from the collaborative work at the StemCellOW workshop discussion, we have developed an Ontology for Stem Cell Investigations (OSCI) with the aim to incorporate entities from the CL, CLO, OBI, and other ontologies to support the standardization and integration of stem cell knowledge. We have also applied OSCI to analyze the iPSC-based BD studies.

## Methods

### OSCI development

Like CL, CLO, and OBI, OSCI also uses the Basic Formal Ontology (BFO) [9] as its upper-level ontology. OSCI imports all stem cell related terms from CL and CLO. OSCI was developed using the standard ontology development strategy of combining top-down and bottom-up methods. The top-down method works by aligning OSCI with existing reliable ontologies such as CL, CLO, and OBI. The bottom-up method works by developing and applying OSCI to a model and representing specific use cases. The use cases in our current stage of study include iPSC experimental protocol standardization and stem cell-based BD study as described below.

The Ontofox tool [10] was used to extract stem cell related terms from existing ontologies and input them into OSCI. The Protégé-OWL editor [11] was used for manual ontology editing.

Since this is a collaboration among multiple parties, we came to consensus through intensive discussions during the StemCellOW workshop and afterwards in follow-up teleconference meetings and email exchanges.

#### Stem cell investigation metadata collection and OSCI representation

Various metadata from different sources were collected, modeled, and represented in OSCI. The three main resources we used include the Minimum Information About a Cellular Assay for Regenerative Medicine (MIACARM) [12], the LINCS metadata standards [13], and the Eagle-i bioresource (https://www.eagle-i.net/).

##### Use case 1: iPSC study for bipolar disorder investigation

OSCI was used to model the pipeline and variables found in the generation of iPSCs and the uses of iPSCs derived from BD patients or healthy human subjects, and supports studies of mechanisms involved in BD. The University of Michigan human Pluripotent Stem Cell Core (hPSCC) directed by Dr. Sue O’Shea offers training in human ESC and iPSC culture and differentiation, and provides advice, technical help and reagents for the generation of new iPSC lines (https://cores.research.umich.edu/core/pluripotent-stem-cell-core/). The studies and use cases taken from the hPSCC provide key examples for OSCI ontology-based stem cell investigation studies.

##### Use case 2: literature mining of bipolar disorder-related stem cell gene markers and their representation in OSCI

To identify genes associated with different stem cell stages (SCSs) we used our PubMed abstract text mining pipeline that we generated for the development of the Molecular Biology of the Cell Ontology (MBCO) [14]. We defined the SCS stem cells derived from patients with bipolar disorder and downloaded all abstracts that were obtained by the following PubMed query:

((“Stem cell”) OR (“Stem cells”) OR (iPSC)) AND (“Bipolar disorder”)

Downloaded abstracts and all other abstracts in our background set were screened for gene related terms. The background set consists of abstracts that we downloaded for the generation of the MBCO (~ 2. 1 million abstracts) and included the abstracts of bipolar disorder SCS. We used Fisher’s Exact test to calculate the selectivity of each obtained gene-SCS association by comparing the number of abstracts in the SCS and background abstract sets that mention or do not mention the gene.

Additional genes were curated from O’Shea and McInnis [8] and added to the predicted gene SCS associations as identified from the literature mining process described above.

The gene markers and their associated processes are modeled and represented in OSCI, with the aim to logically represent and better understand the mechanisms of iPSC formation and applications.

#### OSCI ontology and code access

The OSCI ontology and example SPARQL code is publicly available on the GitHub website: https://github.com/stemcellontologyresource/OSCI. OSCI has been deposited in Ontobee [15] at: http://www.ontobee.org/ontology/OSCI.

## Results

### Modeling stem cells using CL, CLO, and OSCI

**Fig. 1:**
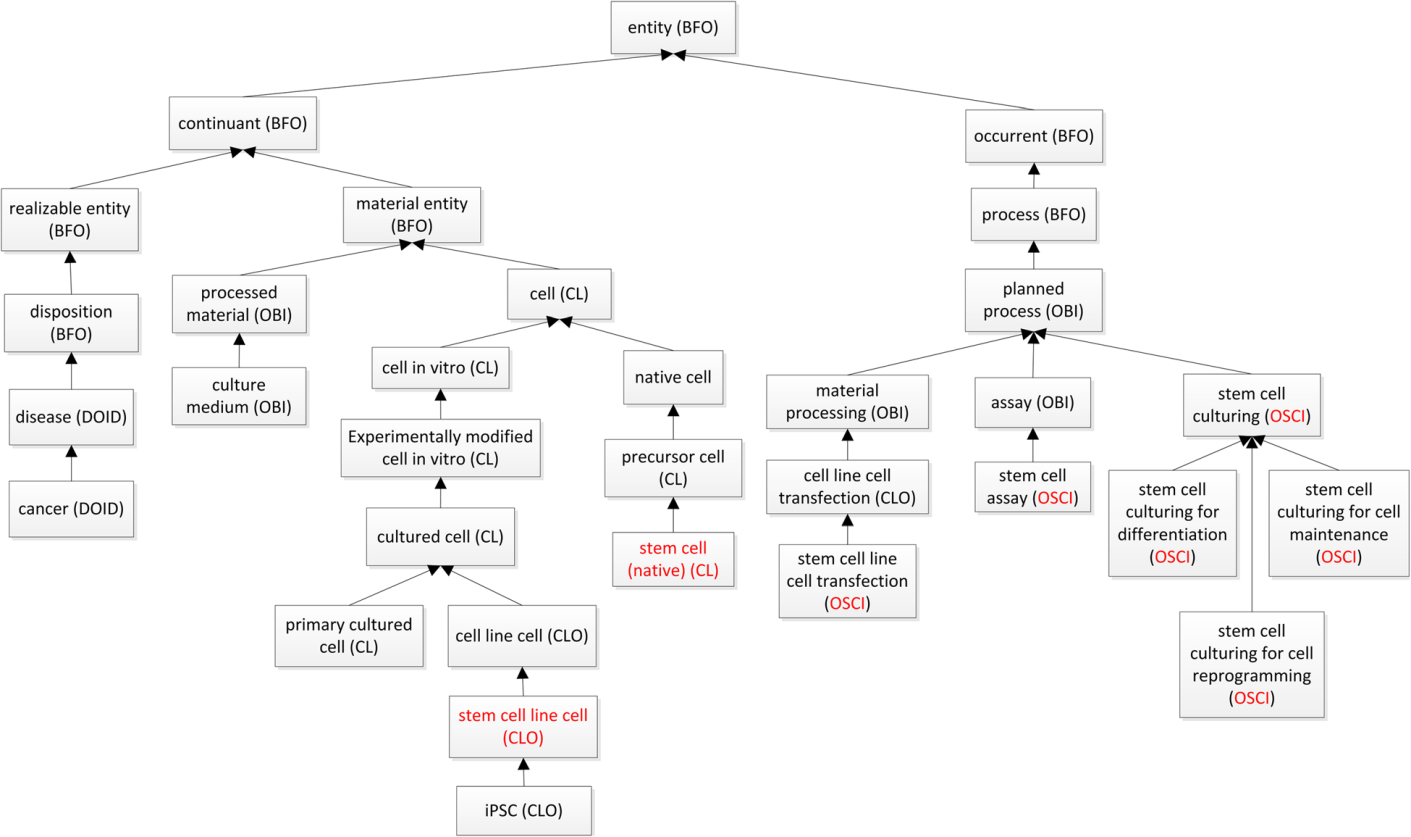
Selective top-level terms and hierarchy of OSCI. Each arrow in the figure represents an ‘is a’ relation where the bottom entity type is a subtype of the entity type above

OSCI is developed as an application ontology to support the collaborative and standardized representation, integration, and analysis of various stem cells in vivo and in vitro. Figure 1 shows the selected upper-level terms and hierarchical structure of the OSCI ontology. OSCI reuses many terms from CL and CLO. CL primarily represents native stem cells, while CLO primarily represents stem cell line cells that have been generated and cultured in vitro and maintain the features of in vivo stem cells. OSCI imports these terms from CL and CLO and aligns them naturally under the same common ontology framework (Fig. 1).

In addition to the imported native stem cell types from CL and stem cell line cell types from CLO, OSCI emphasizes the standardization of stem cell investigation-related metadata types and minimal information standardization. To support this, we have also imported many basic investigation related terms from the OBI, the Ontology of Genes and Genomes (OGG) [16], and the Protein Ontology (PRO) [17]. CLO also includes many cell line cell culture related terms, which are also imported into OSCI.

**Fig. 2:**
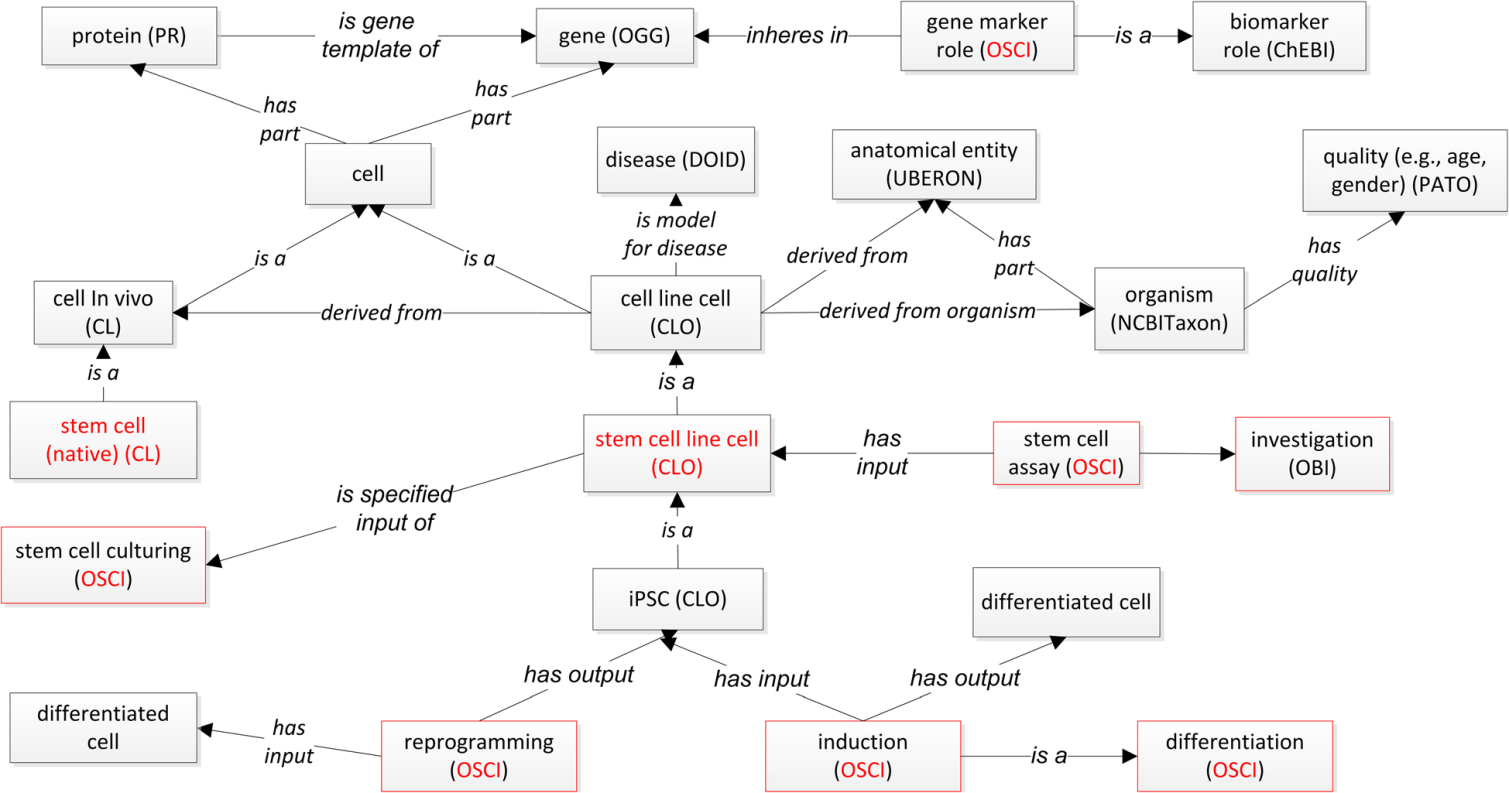
OSCI ontology design pattern. The red boxes represent processes. The names (e.g., OSCI) within parentheses are ontology names. Stem cell and stem cell line cell are two key terms and highlighted in red

Figure 2 shows the general OSCI representation of stem cell investigation with associated experimental variables that are semantically linked together. Several stem cell specific process terms, e.g., stem cell culturing, stem cell assay, reprogramming, are laid out in this Fig.

A total of 193 terms from CL were imported into OSCI. In CL, ‘stem cell’ (CL_0000034) is defined as: “A relatively undifferentiated cell that retains the ability to divide and proliferate throughout life to provide progenitor cells that can differentiate into specialized cells.” Therefore, a stem cell has two fundamental capabilities: self-renewal (i.e., indefinite division while remaining in an undifferentiated state), and the ability to differentiate. These two capabilities are defined in CL as two axioms:

*‘capable of’ some ‘stem cell division’.*

*‘capable of’ some ‘cell differentiation’.*

**Fig. 3:**
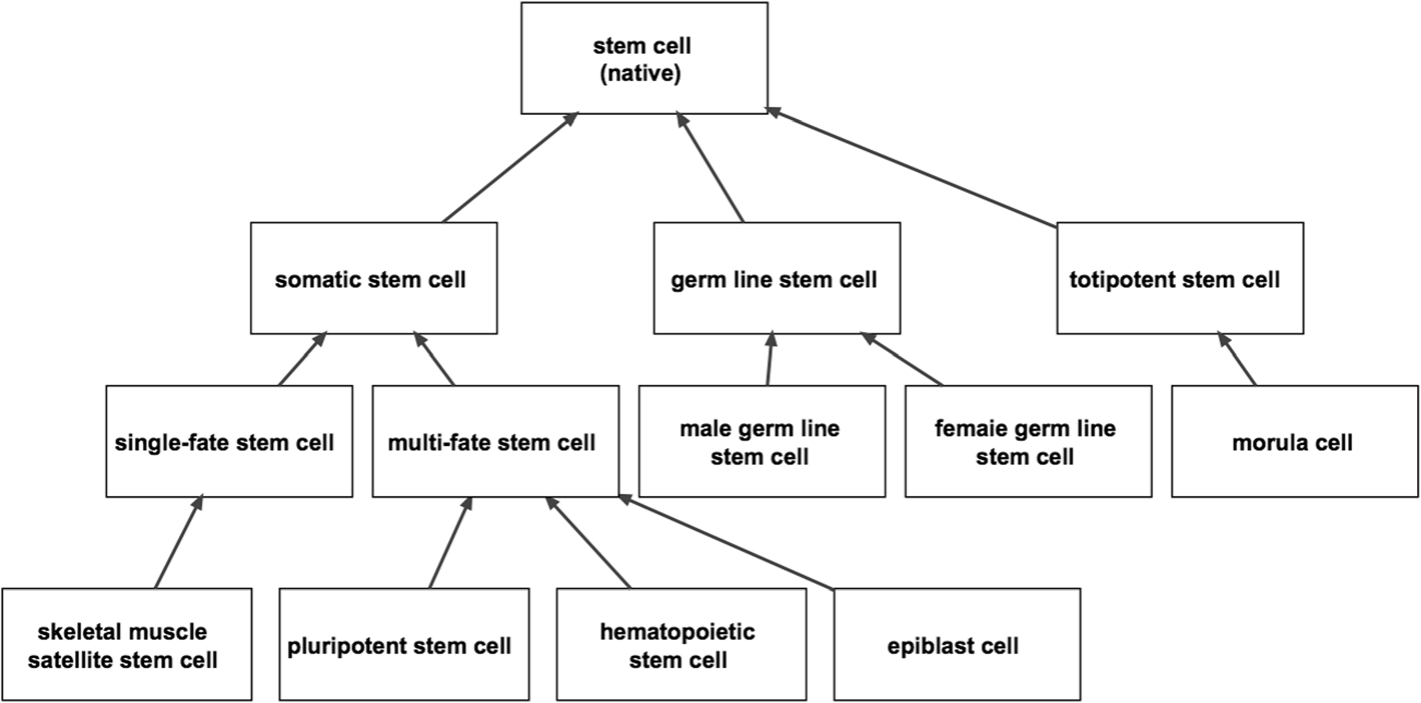
Upper level hierarchy of the CL showing stem cell and examples of specific stem cell subtypes

Figure 3 shows the upper level hierarchy of stem cell terms in the CL. Stem cell terms in the CL are subtypes of ‘native cell’ and represent stem cells as they occur in vivo, in both developmental and mature stages of an organism. The basic division occurs between somatic and germ line stem cells which are differentiated from the totipotent stem cells of the morula from which all cell types arise, including somatic cells, germ cells, and extraembryonic cells such as those of the placenta.

In CLO, a cell line cell is defined as a cell of a stable and homogeneous population (i.e., cell line) of cells with a common biological origin and propagation history in culture. A stem cell line cell is defined in CLO as a cell line cell that has the two capabilities of stem cells, i.e., self-renewal and the ability to differentiate. Notably, these two capabilities of stem cell line cells are the same as the two capabilities for native stem cell defined in CL.

**Fig. 4:**
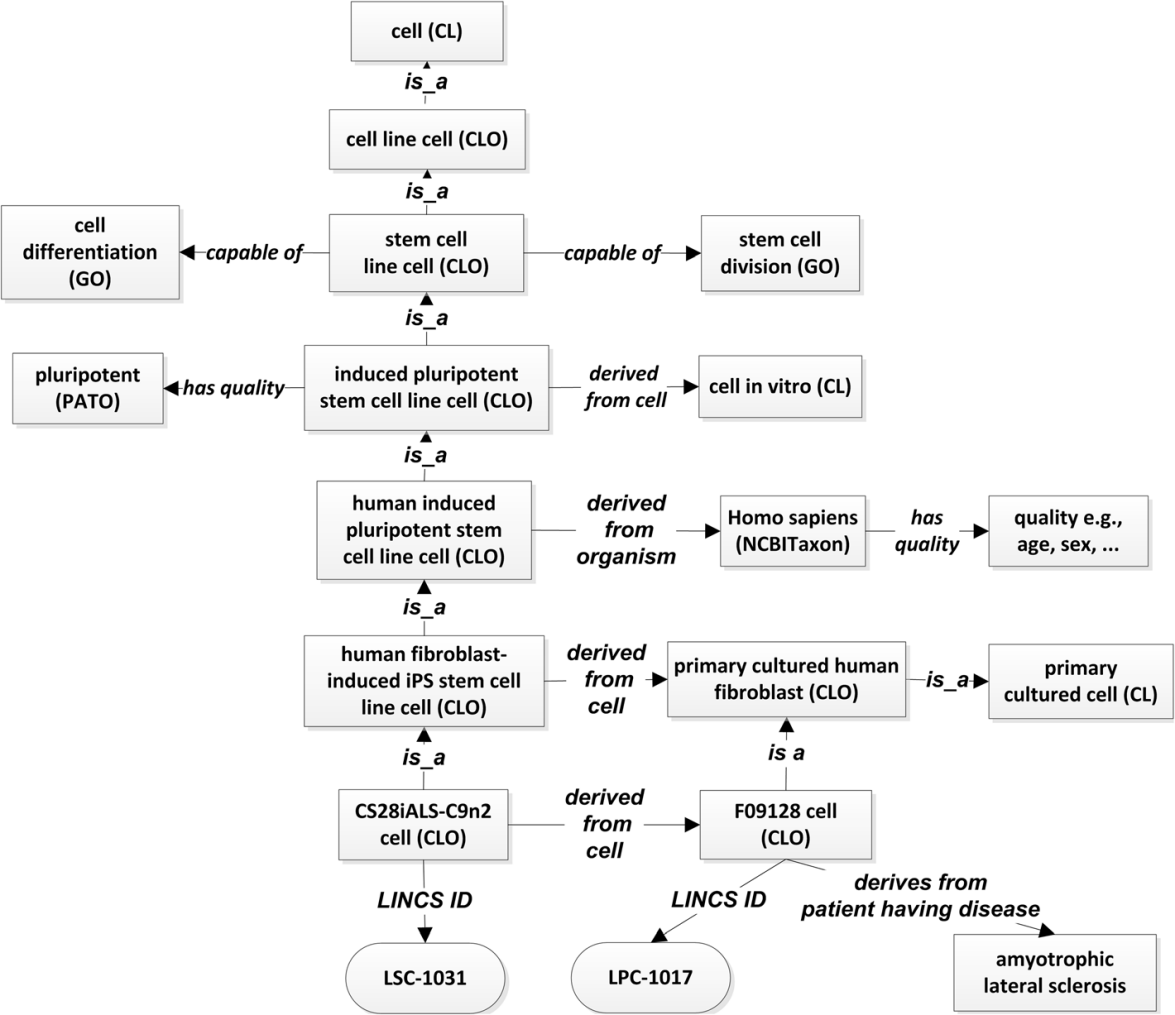
CLO/OSCI design pattern of stem cell line with a LINCS iPSC example. Each square box represents a class term in CLO. The two round cornered terms are annotation text, representing the LINCS IDs for corresponding cell

Figure 4 shows the design pattern of how CLO represents stem cell line cells. Note that CLO represents these stem cell line cells as individual cells instead of as a population (i.e., a ‘line’). OSCI imports the whole branch of stem cell line cells and their semantically linked terms from CLO.

In total, OSCI imports 93 terms from CLO, including 38 stem cell line cell types that are being studied and analyzed in the LINCS project. These stem cell line cells include iPS cell types, embryonic stem cell line cells, and neural stem and progenitor cell line cells differentiated from iPSCs. Approximately 230 unique batches of those cells have been used throughout LINCS assays. Figure 4 presents the example of the CS28iALS-C9n2 cell, a fibroblast-derived iPS cell line cell, to illustrate the CLO design pattern for stem cell line cells.

In addition, OSCI represents different components in various stem cell related processes such as stem cell culturing, reprogramming, differentiation, and assays (Fig. 2).

### OSCI modeling and representation of stem cell experimental investigation metadata types

One of the main goals in developing OSCI is to support ontological representations of stem cell investigation-related metadata types and their relations captured via logical axioms. The selected OSCI metadata terms from two primary resources, the recently reported Minimum Information About a Cellular Assay for Regenerative Medicine (MIACARM) [12] and the LINCS metadata standards [13]. MIACARM describes minimal information required for advanced cellular experiments with human cell types, with a specific emphasis on stem cells. In total, MIACARM includes approximately 130 metadata types, covering the areas of stem cell production, ethical operation, materials (e.g., donor information, source cell, cell culture medium and substrate), the cell banking process, cell characterization, and sterility testing [12].

Furthermore, OSCI includes many metadata fields generated from the LINCS project. The LINCS project has generated metadata standards and data exchange specifications to describe, model, and integrate complex and diverse high-throughput cellular response data [13]. The LINCS metadata specifications cover all manner of biomedical reagents, including small molecules, protein perturbagens, embryonic stem cells, iPS cells, cell lines, primary cells, and more [13].

While other resources, such as the Eagle-i ontology (https://www.eagle-i.net/) also include a reagent registration system and key terms relevant to stem cell general information and experimental design, LINCS and MIACARM were the major sources of metadata terms for the OSCI, because of their well-developed lists of terms focused on cell biology experiments.

The OSCI aims to ontologically represent and standardize these metadata types. We have merged the metadata lists from the aforementioned resources, identified and imported many of these metadata terms from existing ontologies where possible, and generated new terms if we could not find the terms from existing ontologies. Table 1 summarizes the (unique) kinds of information currently collected in the MIACARM and LINCS standards. Most of these terms are already available in OBO Foundry ontologies and, following OBO Foundry principles, we imported terms when possible.

**Table 1:** Stem cell metadata types from MIACARM and LINCS

*Topic*	*Example terms*	*# of terms*
stem cell production information	stem cell name, stem cell id, provider contact information	10
ethical compliance/regulation	informed consent from donor	2
materials used	basal medium, feeder cell name, HLA type	45
cell banking process	source cell transferring protocol, material transferring method	37
stem cell quality control	viability at thawing, morphology, tumorigenesis	24
Total		118

Table 2 summarizes the ontologies from which we imported terms. For brevity, we only list those ontologies from which we imported 50 or more terms. Although not listed, it is important to note that we use the Basic Formal Ontology (BFO) as our upper level ontology. BFO plays a critical role semantically integrating terms from multiple ontologies.

**Table 2:** OSCI terms imported from external ontologies

*Ontology*	*Example terms*	*# of terms*
UBERON (anatomical entities)	organ, tissue	507
Cell Ontology	cell, stem cell, embryonic stem cell, hematopoietic stem cell	193
Ontology for Biomedical Investigations	measurement datum, assay	157
Gene Ontology	cell development, cell differentiation	103
Cell Line Ontology	cell line, cell line repository, induced pluripotent stem cell line cell	82
Protein Ontology	protein, CD14 molecule	54

When a suitable OBO Foundry term was not found, we created specialized OSCI terms. This resulted in the creation of 34 terms whose URIs have the “OSCI_” prefix. Table 3 summarizes the kinds of terms added.

**Table 3:** Newly added OSCI terms

*OSCI Term Type*	*Example terms*	*# of terms*
protocols for passaging and culturing	cell culture protocol, feeder cell preparation protocol	7
production and provider information	provided by, lot number, contact information	7
cell culture media or substrate	cell culture medium additive, feeder cell, TeSR	7
stem cell marker processes	iPSC marker process, neural stem cell marker process	6
donor information	donor screening method, age of donor at time of donation	4
cell passaging and concentration	cell culture passage number, concentration measurement datum	2
publication information	reference publication	1

Many of the OSCI terms provide the basic information about the stem cell culturing and reprogramming processing. In 2012, Shinya Yamanaka was awarded the Nobel Prize in Medicine for his discovery of the generation of iPSCs using 4 transcription factors: Sox2, Oct4, Klf4 and c-Myc, under regular stem cell culture conditions [18]. For regular stem cell culturing, we need: (i) Basal medium, such as Essential 8 (E8) medium or mTeSR1™ medium; (ii) Growth additives (e.g., fetal bovine serum (FBS), leukemia inhibitory factor (LIF)) to maintain cell pluripotency; and (iii) Substrate or matrixes, including feeder cells such as mouse embryonic fibroblasts (MEFs) or an extracellular matrix such as Matrigel™ (or GelTrex and Vitronectin) [19]. For reprogramming, we need the stem cell culturing conditions as well as the presence of specific transcription factors. The Y5 plasmids (pCE-hOCT3/4, pCE-hSK, pCE-hUL, pCE-mp53DD, and pCXB-EBNA1), for example, provide a way to induce expression of key transcription factors.

**Fig. 5:**
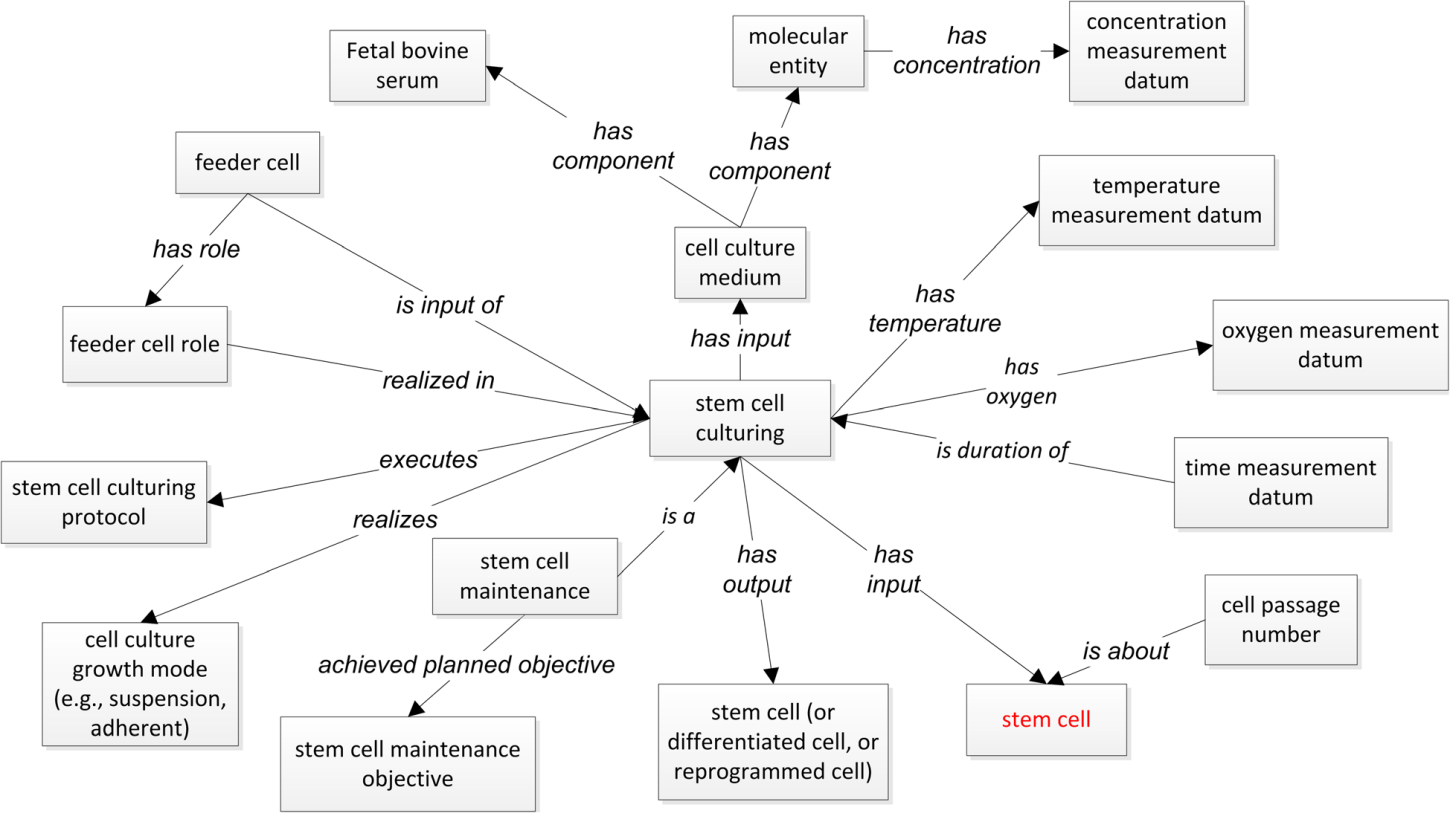
OSCI Stem cell culturing model

Our study identified many important variables and procedures to model and represent in OSCI. Figure 5 illustrates an OSCI modeling of the stem cell culturing, which may be altered in a myriad of ways. More specifically, the process of stem cell culturing needs to consider different experimental conditions including cell culture medium, temperature, culturing time duration, CO_2_, and cell passage number. Likewise, the stem cell culture medium includes a variety of components such as fetal bovine serum (FBS), and molecular entities such as leukemia inhibitory factor (LIF), each at its optimal concentration. One special aspect of stem cell culturing is the optional usage of feeder cells which provide converted nutrients for the growth of stem cells, primarily pluripotent stem cells. While this methodology is less commonplace in most recent stem cell research, the usage of feeder cells is still found in many laboratories as part of stem cell culturing. In OSCI, a cell can serve in the role of a feeder cell (Fig. 5).

Additionally, OSCI provides an ontological platform to represent different protocols of stem cell culturing, which can achieve different objectives, including the maintenance, differentiation, or reprogramming of stem cells (Fig. 5). For example, stem cell maintenance has the objective of maintaining the pluripotency of stem cell (Fig. 5). Cells can be induced or converted from somatic cells to become pluripotent cell (reprogramming), from differentiated cells into progenitor cells (dedifferentiation), and from one type of cell into another cell type (transdifferentiation) [20]. There are also many cell-based assays including identification of stem cells, characterization of stem cells, and phenotyping of stem cells. These processes are defined logically in OSCI.

#### Use case 1: OSCI modeling and representation of bipolar disorder specific stem cell experimental investigation

As a use case study, we applied OSCI to model a stem cell specific use case derived from Dr. O’Shea’s laboratory. Dr. O’Shea is interested in characterizing iPSC lines from patients with bipolar disorder and undiagnosed controls through SNP/CNV (single nucleotide polymorphism/copy number variation) testing, karyotyping, and qPCR of pluripotency and germ layer markers. Dr. O’Shea and her research group differentiate these iPSCs into neuronal cell types including GABAergic, glutamatergic, and glial cells. Furthermore, they collaborate with several universities to standardize iPSC banking practices.

Within recent years, it has been possible to differentiate neurons and astrocytes from iPSCs derived from individuals diagnosed with BD and controls. Gene expression analyses of iPSCs, neurons and glial cells have identified unique patterns of expression of signaling molecules, transcription factors, and microRNAs in the aforementioned study populations, with the goal of identifying new treatments for BD [21].

**Fig. 6:**
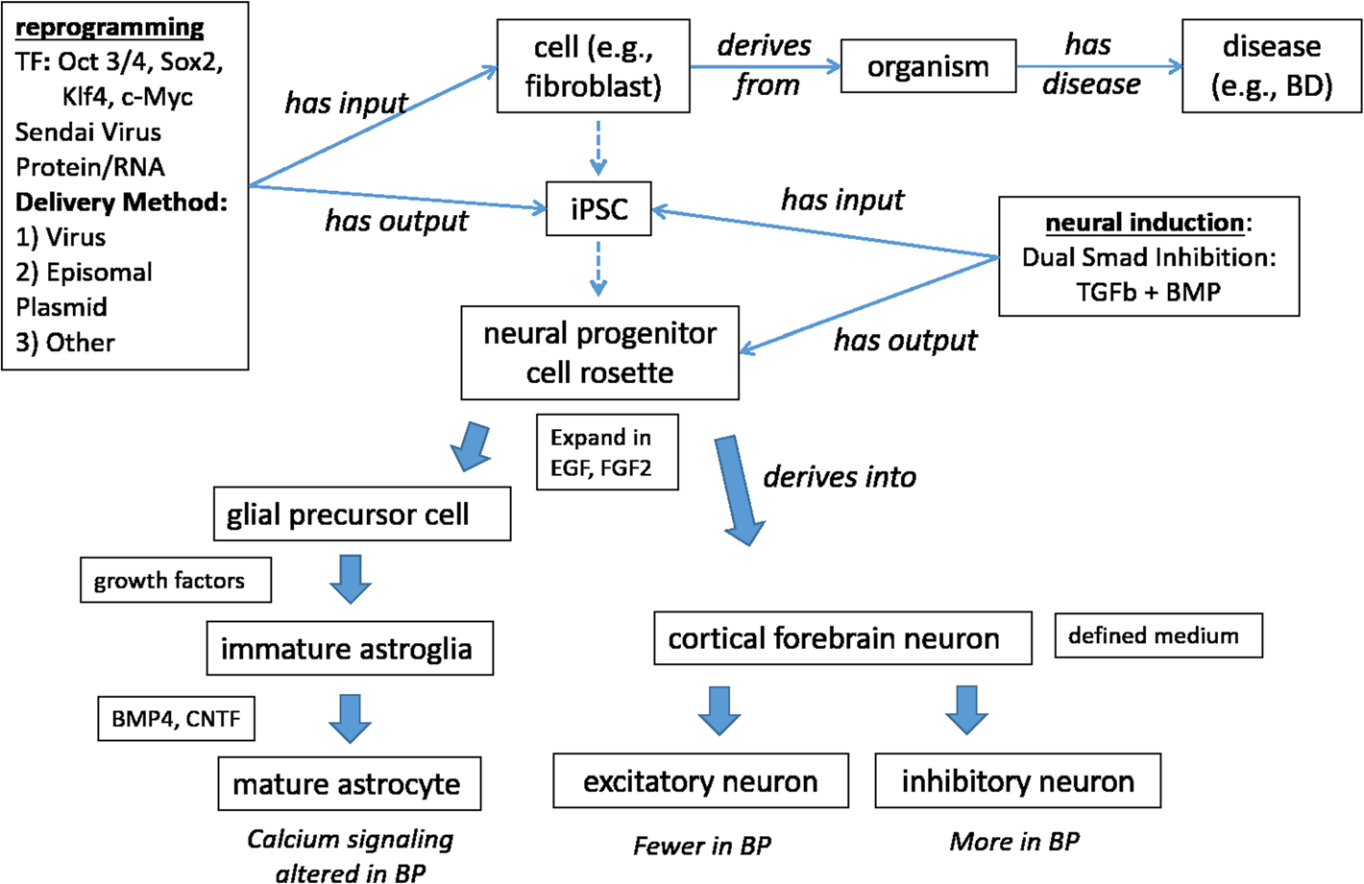
Diagram showing the generation of glial lineage and neuronal lineage cells from various cell types. These cells can then be used to study bipolar disorder (BD). Large blue arrows indicate directed differentiation

Figure 6 illustrates a more detailed procedure of how iPSCs are generated from differentiated cells and then used to study BD. Briefly, fibroblasts, immortalized lymphoblastoid cell lines (LCLs), endothelial cells, and amniotic fluid cells (AFCs) can be reprogrammed to pluripotency using transcription factors (TF): Oct 3/4, Sox2, Klf4, and c-Myc. Factors are delivered by episomal plasmids, virus, or other approaches including PiggyBac constructs. For each of these approaches, knowledge of these factors is important for better representation of the methodological details. For example, for the viral method, we need to identify the multiplicity of infection (MOI) and titer; for episomal reprogramming approaches, it is critical to ensure that there is no integration of the plasmid.

As represented in Fig. 5, neural induction is produced by dual Smad inhibition. Glial Precursor Cells (GPCs) are differentiated into immature astroglia by adding Noggin, PDGFAA, FGF, EGF, LIF, and further matured with BMP4 and CNTF. Neuronal Precursor Cells (NPCs) generate cortical forebrain neurons in the presence of patterning factors. Based on the characteristics of the original cell donors (BD patients or human subject controls), the reprogrammed iPSCs and the iPSC-derived differentiated cell types have different phenotypic profiles. For example, the calcium signaling in mature astrocytes derived from BD patients is altered compared to the cells derived from control humans. Fewer excitatory neurons and more inhibitory neurons can be identified in the BD-derived cells (Fig. 6). Different experimental conditions will also have significant effects on the resulting cell phenotypic profiles. Therefore, it is useful and important to carefully dissect these experimental conditions and model them using an ontological strategy.

The information illustrated in Fig. 6 is also being represented in OSCI. Overall, OSCI provides an ideal ontological framework to logically and systematically represent the details of experimental stem cell studies in a human- and computer-interpretable format.

#### Use case 2: literature mining and modeling of gene markers or alterned genes related to stem cell-based bipolar disorder investigation

Drs. Sue O’Shea (co-author of this paper) and Melvin G. McInnis have recently published a review article that lists 176 genes as gene markers or alterned genes of BD at different stages of cell differentiation including those genes identified in iPSCs and their derived cell types [8]. Many genes in the categories of WNT, Hedgehog or Nodal pathway signaling are altered in BD patients, likely causing the impairment of the differentiation of BD patient-derived neurons to dorsal telencephalic derivatives [8].

Our literature mining using the MBCO algorithm [12] further identified 111 genes that appeared to be related to stem cells and associated with BD. 25 of these genes are mentioned in the review article by O’Shea and McInnis. Manual annotation, using expert knowledge, is currently underway to validate which genes are markers of BD in stem cells (e.g., iPSC) or stem cell-derived cell types. Manual validation will be supported by our pipeline for computer-assisted fast validation [12].

An example of our manual evaluation is the identification of the gene EEF1AP16, i.e., eukaryotic translation elongation factor 1 alpha 1 pseudogene 16 (NCBI Gene ID 387845). Our annotation found that this gene can act in a alterned gene role that is realized in the process of neurodevelopment of iPS cells derived from BD patients [22]. Therefore, we can represent such a relation using the axiom defined below:

EEF1AP16 gene: *‘has role’ some (‘alterned gene role’ and (‘realized in’ some ‘neurodevelopment of iPSC derived from bipolar disorder patient’)).*

We are now in the process of adding all the literature references, and manually verified axioms about gene roles to the OSCI ontology in a logical and ontological way.

### OSCI statistics and query

Currently, OSCI has 1548 terms, including 1310 classes, 103 object properties, 4 data properties, and 113 annotation properties. Following OBO Foundry principles, we imported terms from other OBO Foundry ontologies when possible. Table 2 summarizes the ontologies from which we imported more than 50 classes. Although not listed, it is important to note that we use the Basic Formal Ontology (BFO) [9] as our upper level ontology. BFO plays a critical role semantically integrating terms from multiple ontologies. After importing terms from existing ontologies, we also generated 37 OSCI-specific created specialized terms to represent stem cell investigation specific terms, many of which have been explained earlier in this article. The detailed OSCI statistics can be found at: http://www.ontobee.org/ontostat/OSCI.

The OSCI information can be queried using DL query or SPARQL query (data not shown). With more information added to OSCI, the OSCI query will become a powerful tool to support stem cell and stem cell investigation-related data and knowledge queries and computer-assisted automated reasoning.

## Discussion

This paper reports our development and applications of the OSCI with the aim to integrate, share, and analyze stem cells, including native stem cells and in vitro stem cell line cells. We have also focused our efforts on ontological representation and standardization of metadata data types to support various stem cell investigations.

Our ontology is developed using state-of-the-art technologies and tested with use cases. OSCI reuses multiple subject ontologies all sharing a common upper level ontology (BFO) [9]. The majority of classes in OSCI were extracted from the constituent ontologies rather than having to create classes specific to this endeavor. OSCI is developed as an ontology for use in other stem cell related investigations and is available as a publicly accessible resource, which is consistent with the extensive set of biomedical ontologies of the OBO Foundry [4]. We also utilized semantic search technology, big data and the analysis of unstructured data to correlate terms, gene identifiers, and topics from our use case study related to BD in the development of OSCI. As another use case, we also performed SCP gene marker literature mining and use OSCI to ontologically model the results obtained from the literature mining.

There exist many potential uses for OSCI. For example, OSCI, in conjunction with the CL and CLO, can be used to identify features of stem cell line cells and link these cells based on their features. These ontologies can also be used to identify the origins of a stem cell line, including the initial cell type, tissue, organ, organism, or disease model from which a stem cell line cell was derived. Additionally, OSCI enables querying across multiple sets of data. For example, across two experiments, we can query which factors are shared or differ. As we acquire more use cases, OSCI allows us to highlight commonalities and hone in on the most important factors in these investigations. OSCI can also support many applications from different projects, such as LINCS and the Harvard Stem Cell Commons projects.

In the future, we plan to implement other supplementary features such as additional cell markers, genetic modifications (e.g., gene mutation) and methods of conditioning cell cultures for cell differentiation (e.g., virus transfection). These features then can also be co-analyzed and studied. We will also link the CL/CLO/OSCI cell information to LINCS or other data sets.

## Conclusion

We have developed the Ontology for Stem Cell Investigations (OSCI) to support standardized representation of stem cells in vitro and in vivo and annotation of multiple use cases in stem cell research including describing experimental methodologies, literature mining to identify genes involved in stem cell biology, and integration of data about stem cells and stem cell differentiation.
